# Annual Report of the Postmaster-General of the United States

**Published:** 1891-03

**Authors:** 


ANNUAL REPORT OF THE POSTMASTER-GENERAL OF THE UNITED STATES FOR THE FISCAL YEAR ENDING JUNE 30th, 1890. Washington: Government Printing Office, 1890.

According to this report the total receipts of the Post-Office are nearly $61,000,000. The gross revenue is nearly $5,000,000 larger than it ever was before.

A most convincing argument is made for a system of postal telegraph service. This special improvement has been strenuously advocated by Mr. Wanamaker ever since he came to the office of Postmaster-General, and has made him distinguished.

His second pet scheme is the establishment of postal savings banks. This scheme is warmly advocated in this report.

The third alteration in the management of the Post-Office, for which Mr. Wanamaker has brought to himself general attention, is the limiting of the number of sample copies sent out by periodicals. This is designed to stop the practice of issuing advertisements ostensibly as regular journals, and entering them in the Post-Office as second-class matter at pound rates, and so evading the payment of proper rates of postage.

